# P24. Aviscumine enhances NK- cytotoxicity against tumor cells

**DOI:** 10.1186/2051-1426-2-S2-P15

**Published:** 2014-03-12

**Authors:** G Gamerith, A Amann, B Schenk, T Auer, JM Huber, K Cima, H Lentzen, J Löffler-Ragg, H Zwierzina, W Hilbe

**Affiliations:** 1 Innsbruck Medical University, Clinic of Internal Medicine V, Innsbruck, Austria; 2 Innsbruck Medical University, Department of General and Surgical Intensive Care, Innsbruck, Austria; 3 Innsbruck Medical University, Department of Radiology, Innsbruck, Austria; 4 Innsbruck Medical University, Clinic For Internal Medicine Vi, Innsbruck, Austria; 5 Cytavis Biopharma Gmbh, Innsbruck, Austria

## Background

The mistletoe lectin I belongs to a new class of anticancer drugs with type II ribosomal inhibitor activity. The recombinant mistletoe lectin (aviscumine) has shown immunomodulatory and cytotoxic activity in preclinical models as well as potential antitumor effects in phase I and I/II clinical trials. The aim of this study was to further elucidate the immunostimulatory capacity of aviscumine on natural killer (NK) cell function in a human ex-vivo model.

## Methods

The effect of aviscumine (0.5 and 1 ng/ml) on the cellular cytotoxicity of NK cells isolated from peripheral blood mononuclear cells (PBMCs) of 34 healthy volunteers was measured via a standard Chromium^51^ release assay against K562 chronic myelogenous leukemia cells. For further validation changes in expression of the NK cell activation marker CD107α was determined via flow cytometry (FACS) in 13 volunteers.

## Results

Aviscumine induced a significant concentration-dependent increase in NK cellular cytotoxicity in about 54% of the volunteers (p<0.001). This enhancement was also observed with low dose IL-2 stimulation (p=0.01). FACS analysis revealed an aviscumine triggered up-regulation of the NK cell degranulation marker CD107a (p=0.001).

## Conclusion

Functional ex-vivo analysis of NK cells from healthy donors revealed a direct immune stimulatory mechanism of aviscumine. These data further strengthen its potential as immunomodulatory antitumor agent and suggest that NK cell activity in peripherial blood may be evaluated as predictive biomarker in clinical trials.

